# Consensus of Minimally Invasive and Multidisciplinary Comprehensive Treatment for Hepatocellular Carcinoma – 2020 Guangzhou Recommendations

**DOI:** 10.3389/fonc.2021.621834

**Published:** 2021-07-02

**Authors:** Qi-Feng Chen, Wang Li, Simon Chun-ho Yu, Yi-Hong Chou, Hyunchul Rhim, Xiaoming Yang, Lujun Shen, Annan Dong, Tao Huang, Jinhua Huang, Fujun Zhang, Weijun Fan, Ming Zhao, Yangkui Gu, Zhimei Huang, Mengxuan Zuo, Bo Zhai, Yueyong Xiao, Ming Kuang, Jiaping Li, Jianjun Han, Wei Song, Jie Ma, Peihong Wu

**Affiliations:** ^1^ Department of Medical Imaging and Interventional Radiology, Sun Yat-sen University Cancer Center, Guangzhou, China; ^2^ State Key Laboratory of Oncology in South China, Sun Yat-sen University Cancer Center, Guangzhou, China; ^3^ Collaborative Innovation Center for Cancer Medicine, Sun Yat-sen University Cancer Center, Guangzhou, China; ^4^ Department of Imaging and Interventional Radiology, The Chinese University of Hong Kong, Hong Kong, China; ^5^ Department of Medical Imaging and Radiological Technology, Yuanpei University of Medical Technology, Hsinchu, China; ^6^ Department of Radiology, Taipei General Hospital and School of Medicine, National YangMing University, Taipei, China; ^7^ Department of Radiology, Yeezen General Hospital, Taoyuan, China; ^8^ Department of Radiology and Center for Imaging Science, Samsung Medical Center, Sungkyunkwan University School of Medicine, Seoul, South Korea; ^9^ Image-Guided Bio-Molecular Intervention Research and Division of Vascular and Interventional Radiology, Department of Radiology, University of Washington School of Medicine, Seattle, WA, United States; ^10^ Department of Surgery, Shanghai Jiaotong University School of Medicine Renji Hospital, Shanghai, China; ^11^ Department of Radiology, The First Medical Centre, Chinese People’s Liberation Army (PLA) General Hospital, Beijing, China; ^12^ Department of Liver Surgery, The First Affiliated Hospital of Sun Yat-sen University, Guangzhou, China; ^13^ Department of Interventional Oncology, The First Affiliated Hospital, Sun Yat-sen University, Guangzhou, China; ^14^ Department of Intervention, Shandong Cancer Hospital, Jinan, China; ^15^ Department of Oncology, Shandong Provincial Hospital Affiliated to Shandong First Medical University, Jinan, China; ^16^ Department of Biotherapy, Beijing Hospital, National Center of Gerontology, Chinese Academy of Medical Sciences & Peking Union Medical College, Beijing, China

**Keywords:** hepatocellular carcinoma, minimally-invasive therapy, multidisciplinary comprehensive treatment, consensus, Guangzhou recommendations

## Abstract

In China, the majority of patients with hepatocellular carcinoma (HCC) result from long-term infection of hepatitis B. Pathologically, HCC is characterized by rich blood supply, multicentric origins, early vascular invasion and intrahepatic metastasis. Therefore, HCC is not a local disease but a systemic disease at the beginning of its occurrence. For this reason, a comprehensive treatment strategy should be adopted in the management of HCC, including local treatments (such as surgical resection, radiofrequency ablation, microwave ablation, chemical ablation and cryoablation, etc.), organ-level treatments [such as transcatheter arterial infusion of chemotherapy and transcatheter arterial chemoembolization (TACE)], and systemic treatments (such as immunotherapy, antiviral therapy and molecular targeted therapy, etc.). This consensus sets forth the minimally-invasive and multidisciplinary comprehensive guideline of HCC, focusing on the following eight aspects (1) using hepaticarteriography, CT hepatic arteriography (CTHA), CT arterial portography (CTAP), lipiodol CT (Lp-CT), TACE-CT to find the intrahepatic lesion and make precise staging (2) TACE combined with ablation or ablation as the first choice of treatment for early stage or small HCC, while other therapies are considered only when ablation is not applicable (3) infiltrating HCC should be regarded as an independent subtype of HCC (4) minimally-invasive comprehensive treatment could be adopted in treating metastatic lymph nodes (5) multi-level subdivision of M-staging should be used for individualized treatment and predicting prognosis (6) HCC with severe hepatic decompensation is the only candidate criterion for liver transplantation (7) bio-immunotherapy, traditional Chinese medicine therapy, antiviral therapy, and psychosocial and psychopharmacological interventions should be advocated through the whole course of HCC treatment (8) implementation of multicenter randomized controlled trials of minimally-invasive therapy versus surgery for early and intermediate stage HCC is recommended.

## Introduction

Primary liver cancer is the fourth most common malignant tumor and the third leading cause of cancer death in China, which seriously threatens the lives and health of Chinese people. Hepatocellular carcinoma (HCC) accounts for 85% to 90% of primary liver cancer (1, 2). In China, chronic hepatitis B virus (HBV) infection is the main cause of HCC. Approximately 85% of HCC cases are associated with HBV infection, and only approximately 10% are associated with hepatitis C virus (HCV) infection. Conversely, approximately 70% of HCC cases in European countries, North America, and Japan are associated with alcohol and HCV infection (1, 3). China has a large population of patients with liver cancer. There is not yet a worldwide consensus on the treatment strategy for liver cancer, and there is significant divergence in the guidelines for liver cancer treatment in different countries. The epidemiological characteristics, pathogenesis, biological behaviors, staging, diagnosis, treatment, and prognosis of HCC in China are significantly different from those in Europe, North America, and Japan. Therefore, the HCC guidelines developed by the Liver Disease Associations of the European countries, North America, and Japan are not fully applicable to HCC diagnosis and treatment in China. Even the “guidelines or expert consensus” representing the opinions of professionals in different disciplines or different societies are controversial in China. In China, the vast majority of HCC cases result from long-term HBV infection and cirrhosis development. Pathologically, HCC is characterized by a rich blood supply and multicenter origins, with early invasion of small branches of the portal vein and intrahepatic metastasis. Therefore, HCC is not only a local organ disease but also a systemic disease from the beginning of its occurrence (4). In recent years, with the continuous advancement of minimally invasive interventional treatment techniques for HCC guided by imaging and studies of related large-scale randomized clinical trials, the efficacy of minimally invasive interventional therapy has been enhanced. In the meantime, the benefits of a multidisciplinary comprehensive treatment regimen for HCC have also been widely recognized in the clinic. In 2015, Minimally Invasive Therapy in Oncology of Chinese Anti-Cancer association published an article in the “National Medical Journal of China” (also called “Zhonghua Yi Xue Za Zhi”) to preliminarily describe the strategy for minimally invasive, multidisciplinary and comprehensive diagnosis and treatment of HCC (5). On the basis of the preliminary strategy, this consensus further summarizes previous achievements and experience in HCC treatment and highlights the following trends in HCC treatment: 1. More accurate diagnosis and staging; 2. Interventional and minimally invasive treatment, biological immunotherapy, Chinese herbal medicine, psychosocial and psychopharmacological interventions, and humanistic care, which constitute the basic framework for a modern HCC treatment approach; 3. Further explanation of the “constructive treatment concept and strategy” for tumors consistently advocated by the authors, i.e., while effectively inactivating the tumor, the physiological functions, immune function, and quality of life of the patients are optimally preserved. When choosing treatment strategies and methods, minimally invasive interventional therapy combined with multidisciplinary comprehensive treatment is preferred, and extensive wound damage should be avoided or reduced as much as possible. This consensus integrates the clinical diagnosis and treatment strategies of HCC in China and aims to reflect the individualized, rational, and humanistic features of a constructive treatment regimen for HCC.

## Diagnosis

HCC diagnosis comprises two major aspects: clinical diagnosis and pathological diagnosis. Clinical diagnosis primarily depends on determination of cirrhosis history caused by chronic hepatitis (HBV and/or HCV) infection and/or other causes, serological diagnosis, and imaging diagnosis.

### Serological Diagnosis

More than 60% of HCC patients in China show serum alpha-fetoprotein (AFP) levels >400 ng/ml. Therefore, AFP is of significant importance for surveillance and diagnosis of HCC in China (6). For patients with AFP≥400 µg/L for more than 1 month, ≥200 µg/L for 2 months, or a gradually increased and stabilized AFP level but without pregnancy, gonadal embryoma, or active liver disease, HCC should be highly suspected. However, notably, when the AFP level is normal or below the diagnostic criteria, HCC cannot be completely excluded. Approximately 30% of patients with HCC have AFP levels below 20 ng/mL, and 10% to 42% of AFP abnormalities are caused by pregnancy, gonadal embryoma, active hepatitis, the active inflammatory stage of cirrhosis, or metastatic liver tumors (7). Therefore, AFP cannot be used as the only indicator for HCC surveillance and diagnosis. At present, many studies have found that des-γ-carboxy prothrombin (DCP) [also known as protein induced by vitamin K absence or antagonist (PIVKA) II] and AFP-L3/AFP assessments can improve the sensitivity and specificity of early liver cancer diagnosis. DCP>40 mAU/mL or AFP-L3/AFP>15% suggests the possibility of liver cancer (8). Application of the methylation spectrum of circulating tumor DNA (ctDNA) in the diagnosis of tumors, which is one of the “liquid biopsy” markers, is a hotspot in the field of cancer research using circulating tumor nucleic acids. Xu et al. (9) examined the methylation level of specific loci on ctDNA using a few milliliters of blood for early diagnosis of HCC and achieved a diagnostic sensitivity of 84.8% and a specificity of 93.1%.

### Imaging Diagnosis

Imaging plays a crucial role in HCC diagnosis. Currently, the imaging examination methods used for HCC diagnosis primarily include ultrasound, computed tomography (CT), magnetic resonance imaging (MRI), digital subtraction angiography (DSA), and positron emission tomography (PET)/CT. Dynamic enhanced CT and/or MRI are the main diagnostic tools for HCC. CT arterial portography (CTAP)/CT hepatic arteriography (CTHA) combined with lipiodol CT (Lp-CT) can improve the sensitivity and specificity of HCC diagnosis (especially for lesions with a diameter <1 cm). However, since CTAP, CTHA, and Lp-CT are invasive diagnostic procedures, they are used as secondary imaging-based diagnostic methods for HCC. Ultrasound examination is easily available and convenient to perform for initial HCC screening, but the results tend to be affected by the skill of the operators, equipment, liver texture, patient’s body built, obstacles from bone and air. In addition, cholangiocarcinoma tends to contribute to false positive findings in contrast-enhanced ultrasound. PET/CT is beneficial in small population for evaluating the extension of HCC. Therefore, ultrasonography and PET/CT are not included in the present HCC diagnostic criteria. Moreover, diagnosis of specific liver cancer types should be emphasized, such as infiltrative and small HCC.

As the primary imaging-based diagnostic method, the characteristic manifestations indicative of HCC diagnosis observed with dynamic contrast-enhanced CT and/or MRI are arterial phase enhancement and washout during the portal venous phase or delayed phase (10, 11). Statistically, the specificity of dynamic enhanced CT and/or MRI for diagnosis of lesions 1-2 cm in diameter with typical nodule manifestations is 96.6%, and the sensitivity is 62%. For nodules with a diameter > 2 cm, the sensitivity reaches 96% (12, 13). Therefore, for nodules with a diameter >1 cm, when dynamic enhanced CT and/or MRI demonstrate arterial phase enhancement and washout during the portal venous phase or delayed phase, HCC should be highly suspected. However, attention should be paid to the specific manifestations of infiltrative HCC. Infiltrative HCC is defined as an HCC lesion with unclear borders diffusely distributed in multiple hepatic segments, occupying an entire hepatic lobe or the entire liver. Infiltrative HCC accounts for 7% to 13% of all HCC cases. It is more commonly seen in patients with HBV infection, frequently associated with portal vein tumor thrombosis, and has a poor prognosis. Infiltrative HCC is often accompanied by cirrhosis and produces nodules similar to cirrhosis, which are difficult to detect with CT and MRI. MRI diagnosis is more meaningful than CT and is based on an uneven, slightly lower signal on T1 WI, a slightly higher signal on T2 WI, limited diffusion, mostly uneven or miliary enhancement in the arterial phase, and no washout in the portal venous phase, which, in contrast, demonstrates continuous enhancement (14, 15). Due to the poor sensitivity of dynamic enhanced CT/MRI in the diagnosis of HCC lesions smaller than 1 cm, the current European and American diagnostic criteria only apply to HCC larger than 1 cm, and the diagnostic value of liver-specific contrast agents is not emphasized in those guidelines. Although liver-specific contrast agents can improve the diagnostic rate of MRI for HCC smaller than 1 cm, the sensitivity is still low (approximately 46%) (16). When HCC is highly suspected in clinical practice, and CT and MRI cannot detect a lesion with a typical imaging manifestations, work-up with combined application of secondary imaging diagnostic methods should be done.

As a secondary imaging diagnostic method, CTAP reveals a HCC on the basis of portal venous blood supply, which is displayed as a filling defect on the background of highly enhanced normal liver tissue. On CTHA, HCC appear as enhanced nodules at arterial phase, which should be distinguished from arterial-portal shunt and abnormal perfusion. CTHA combined with CTAP can reduce the false positive rate and significantly improve diagnostic sensitivity (from up to 80% to 95%) and specificity for HCC (especially lesions with a diameter ≤1 cm) (17). Some studies have found that CTHA/CTAP can detect 32.8% of lesions that are not detected with dynamic enhanced CT, especially in patients with an HBV (-) status, multiple nodules, and intrahepatic recurrence or metastasis after treatment (18, 19). CTHA/CTAP can accurately assess the extent of the lesion and identify disseminated intrahepatic foci. In the meantime, CTHA/CTAP is more sensitive to intrahepatic hemodynamic changes, which is beneficial for assessment of the infiltration status of intrahepatic blood vessels (even tiny blood vessels) (20, 21). Therefore, CTHA/CTAP provides more precise pretreatment tumor staging and facilitates selection of the optimal treatment choice. Clinically, approximately 15% of small HCC lesions or foci cannot be detected by CTHA/CTAP and require combined examination *via* Lp-CT (22–26). The Lp-CT is performed by injecting 3 to 4 mL of lipiodol through the hepatic artery, followed by liver CT 2 weeks later, and HCC is manifested as intrahepatic lipiodol deposition foci. Lipiodol deposits in HCC are generally dense and uniform, and lipiodol deposits in some necrotic HCC are incomplete and distributed in the periphery or center of the lesion. Lp-CT is of significant importance for detection of HCC with a low degree of differentiation, micro-HCC, small lesions with multicenter origins and intrahepatic micrometastases, and lipiodol also has certain therapeutic effects (22, 27, 28). As early as in 2003, Wu and co-editors monographed a book in Chinese “Minimally Invasive and Multidisciplinary Comprehensive Treatment of Hepatocellular Carcinoma” detailed the diagnosis and treatment of micro-HCC (29). Micro-HCC is defined as follows: 1. Lesion diameter ≤ 0.5 cm; 2. Clinically elevated AFP level (AFP ≥ 400 µg/L for more than 1 month or ≥ 200 µg/L for 2 months); 3. Lesions that were detected by CTAP/CTHA or/and present as spotted lipiodol deposits in Lp-CT; 4. AFP decreased or returned to normal level after Lp-CT or transcatheter arterial embolization (TAE). In view of the invasiveness and high cost of CTHA/CTAP and Lp-CT, these examinations are recommended as secondary diagnostic tools or combined with transarterial chemoembolization (TACE) or TAE to facilitate accurate diagnosis and staging and to simultaneously achieve therapeutic goals.

### Pathological Diagnosis

**Figure 1:**
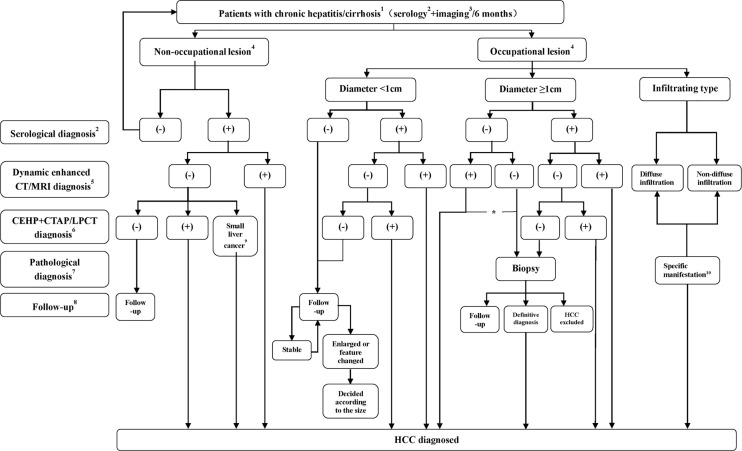
Flowchart of HCC diagnosis. ^1^ The flowchart is mainly applicable to the diagnosis of HCC patients with HBV and/or HCV infection or a liver cirrhosis background related to various causes. For intrahepatic space-occupying lesion patients without hepatitis or a cirrhosis history, diagnosis is usually made by using biopsy; ^2^ or serological diagnosis including AFP, AFP-L3, or DCP detection. Serological diagnosis (**+**) refers to AFP ≥ 400 µg/L for more than 1 month or ≥ 200 µg/L for 2 months, DCP > 40 mAU/mL or AFP- L3/AFP>15%; ^3^ including ultrasound or preliminary CT/MRI examination, with ultrasound more commonly performed; ^4^ No nodule refers to no nodule detected in the liver during imaging follow-up, and intrahepatic nodule refers to a nodular lesion detected in the liver during imaging follow-up; ^5^ Significant enhancement during the arterial phase and washout during the portal venous phase or delayed phase; ^6^ Nodule enhancement in CTHA, filling defect in CTAP and lipiodol deposits in the nodules 2 to 3 weeks after lipiodol injection revealed by Lp-CT; ^7^ Biopsy (-) does not rule out the possibility of HCC, which still requires close follow-up; ^8^ Serological plus imaging monitoring is performed every 3 months during the first 2 years, and the patient is considered stable if there is no significant change and is then followed up at 6 months intervals; ^9^ Micro-HCC is defined as follows: 1. Lesion diameter ≤ 0.5 cm; 2. Clinically elevated AFP (AFP ≥ 400 µg/L for more than 1 month or ≥ 200 µg/L for 2 months); 3. Lesions that were detected by CTAP/CTHA or/and present as spotted lipiodol deposits in Lp-CT or TACE; 4. AFP decreased or returned to a normal level after Lp-CT or TACE; ^10^ Refers to nondiffuse infiltration and diffuse distribution of lesions with an unclear boundary in the liver. T1 WI shows an uneven, slightly low signal, and T2 WI shows a slightly high signal, limited diffusion, and uneven or miliary enhancement or slight enhancement in the arterial phase and no washout in the portal venous phase, which instead would demonstrate continuous enhancement; *For nodules with a diameter of more than 1 cm with typical imaging manifestations, when serological diagnosis is negative, the lesions should be distinguished from primary HCC and other rare pathological types. If the patients choose nonsurgical treatment, a needle biopsy is recommended.

Pathological diagnosis of HCC is recommended when imaging examination shows noncirrhotic liver nodules, uncertain or atypical imaging findings in a cirrhotic liver, or when serology and imaging diagnoses are contradictory. Studies have found that the positive biopsy rate of lesions smaller than 2 cm is only approximately 60% (30), and therefore, negative biopsy results do not completely rule out HCC diagnosis, and still requires further diagnostic work-up or clinical follow-up. **Figure 1** shows the HCC diagnosis process.

## Treatments

HCC occurrence and development are complicated dynamic processes involving multiple factors that are conjoined in multiple steps and include different modifications and gene mutations in many molecular pathways. HCC usually originates from chronic hepatitis, proceeding to cirrhosis and then to HCC. In China, the majority of liver tumors have a rich blood supply, and are accompanied by a background of hepatitis or cirrhosis, and exhibit multicenter occurrence, portal vein tumor thrombus and intrahepatic dissemination even at the its early presentation (4). Therefore, single local treatment cannot achieve a curative effect, and a comprehensive treatment regimen should be adopted that includes local treatment (surgery and ablation) combined with organ level treatment (TACE and perfusion chemotherapy) and systemic treatment (psychotherapy, antiviral treatment, molecular targeting, biological immune therapy, and Chinese traditional medicine treatment).

This consensus emphasizes that minimally invasive treatment should be considered first for early-stage HCC when tumor ablation is safe. If minimally invasive treatment is not applicable for the patient after detailed evaluation, the more traumatic surgical treatment can be considered. The various current treatments and comprehensive treatment principles are described below.

### Local Ablation

Local ablation is minimally invasive, safe, effective, and can be performed repeatedly. Ablation methods include physical ablation (such as radiofrequency ablation (RFA), microwave ablation (MWA), cryoablation, high intensity focused ultrasound (HIFU) ablation, and irreversible electroporation (IRE) ablation) and chemical ablation [such as percutaneous ethanol injection (PEI) or percutaneous acetic acid injection (PAI)]. For single HCC lesion with a diameter ≤ 2 cm, local ablation treatment can achieve long-term efficacy similar to or superior to surgical treatment, with the advantages of less liver function damage, fewer complications, faster recovery, and shorter hospitalization and therefore should be considered the top option (31–33). For a single HCC lesion with a diameter of 3 to 5 cm or 2 to 3 lesions with diameters < 3 cm, the therapeutic effect can be improved by combining TACE treatment with a suitable ablation technique (34). For tumors with a diameter of more than 5 cm or more than three tumor lesions, local ablation cannot inactivate all of the tumor tissue; small satellite lesions are easily missed, and the local recurrence rate is high. Therefore, TACE combined with ablation is significantly superior to ablation alone under these circumstances. A potential drawback is that such combination requires two consecutive treatment sessions, which poses an increased risk for multifocal tumor recurrence or focal progression through repeated manipulation (35). For a tumor to be considered inside the ablation safety zone, the lesion must not be close to or contiguous to the gallbladder, the hilum of the liver, stomach, intestines, or the heart. Ablation of tumors in these dangerous areas may require comprehensive ablation methods, including the water insulation technique (hydrodissection), combined with chemical ablation and particle (^125^I seed) implantation.

RFA and MWA are the most widely used local ablation techniques, with well documented therapeutic effects. When the lesion is larger than 2 cm, RFA is superior to PEI. For tumors adjacent to a large blood vessel or for a large tumor, the effect of MWA may be better due to its shorter ablation procedure time, larger ablation zone, and lower heat sink effect compared with RFA. However, the current RFA and WMA comparison studies show no significant difference in local efficacy and occurrence of complications between the two methods (36–38). Cryospheres formed by cryoablation can be easily observed at imaging (especially CT and MRI) studies, which is convenient for controlling the ablation zone to avoid damaging surrounding normal structures or tissues. However, the incidence of complications, such as hemorrhage, in cryoablation is significantly higher than that in RFA, and thus, cryoablation is not widely used in liver cancer treatment (39, 40). HIFU, by combining non-touch, conformal, and real-time treatment, has advantages for the treatment of multiple tumors and/or liver cancer in some specific locations. However, HIFU treatment takes a longer time, and it is relatively difficult to locate the tumor. Color Doppler ultrasound monitoring can be performed in real-time and is convenient; however, due to the interference of the ribs and gastrointestinal gases, the efficacy of this treatment is dependent on the operator’s experience and skill. Furthermore, the high echo shadow generated during ablation affects observation of the treatment efficacy. HIFU treatment has the deficiencies of therapeutic dosimetry and unclear effects on normal tissues (nonablation areas) after therapeutic ultrasound. Clinically, there is a lack of studies comparing HIFU with other ablation treatments, and thus, the efficacy of HIFU is difficult to evaluate (41–43). As a new nonthermal ablation technique, IRE employs high voltage to irreversibly damage cell membranes and induce apoptosis. IRE treatment has the advantages of a short procedure time, precise ablation zone, no influence of a heat sink effect, and no damage to large blood vessels or bile ducts. Therefore, IRE provides a new option for HCC treatment at certain locations (such as lesions adjacent to large blood vessels and bile ducts and the subcapsular area). However, its effectiveness and safety still await validation in larger cohorts of patients (44–46).

### Surgical Treatment

Surgical treatment is one of the main treatments for HCC and includes liver resection and liver transplantation. For patients in good general condition with a sufficient liver function and remnant liver (15-min retention rate of indocyanine green test <14%), and no severe disease involving the heart, lung, kidney, or other important organs, hepatectomy can be performed. The indications for liver transplantation in patients with HCC are controversial. In China, the incidence of HCC is high, donor livers are not easily available, the cost of transplantation is high, and long-term use of immunosuppressive drugs after liver transplantation is necessary, which leads to inevitable postoperative recurrence. This situation is incompatible with the rapid development and progress in tumor immunology research in the past 10 years. Therefore, liver transplantation is not the first choice or routine treatment for HCC, especially for the treatment of early-stage HCC without cirrhosis decompensation and liver failure.

### Hepatic Artery Interventional Treatments

Trans-arterial interventional treatments for HCC primarily include TAE, TACE, hepatic arterial infusion chemotherapy (HAIC) and transcatheter arterial radioembolization (TARE). Lp-TACE [also known as conventional TACE (cTACE)] has the widest clinical applications.

For patients with permitting liver function, lipiodol-TACE (Lp-TACE) should be the first treatment choice and can accurately stage and detect subcentimeter lesions and achieve organ level treatment. Lp-TACE as the first-line treatment for patients with intermediate stage HCC (2 to 3 lesions with diameters >3 cm or >3 lesions without portal vein tumor thrombus or extrahepatic metastasis) can effectively block the arterial blood supply to the liver tumor and continuously release a high concentration of chemotherapeutic drugs, which result in ischemic necrosis, shrinkage of the tumor, and control of tumor growth while having little effect on normal liver tissue. In Lp-TACE, ultra liquid lipiodol is fully mixed with chemotherapeutic drugs to form an emulsion, which is injected into the tumor blood supplying artery *via* microcatheter superselection. Commonly used chemotherapeutic drugs are anthracyclines and platinum, and combined administration is better than single drug use (47, 48). Enhanced embolization refers to the combination of gelatin sponge embolization with (after) Lp-TACE treatment, which can increase the efficacy of Lp-TACE. Drug-eluting bead (DEB)-TACE employs bead, which can carry a sufficient drug amount and slowly release chemotherapeutic drugs to achieve and maintain a lethal dose in the tumor tissue for several days to several weeks, while the drug concentration in systemic blood circulation is very low. After DEB-TACE treatment, the rate of tumor necrosis is high, and the adverse effects of systemic chemotherapy are mild. Previous study showed that the occurrence of locoregional complications and global hepatic damage after DEB-TACE, such as bile duct injury, intrahepatic biloma, and liver function impairment (presented as high baseline prothrombin value), was significantly higher than that with Lp-TACE alone and was more obvious in patients with severe cirrhosis (49). Moreover, the antitumor effect and overall survival between the two treatments are not significantly different (50–53). It is therefore suggested that Lp-TACE may be more appropriate than DEB-TACE in patients with less advanced cirrhosis.

Lp-TACE can provide diagnostic information and choice of treatment with the following advantages: 1. Induction of necrosis and tumor shrinkage to achieve a downstaging effect and obtain opportunities for surgery or ablation; 2. Detection of missed lesions on other imaging modalities, especially inconspicuous foci scattered small lesions; 3. Reduction of the blood supply inside and around the tumor, thereby reducing the impact of a heat sink effect; 4. Deposited lipiodol is has a positioning effect, thereby improving treatment accuracy (54, 55). For patients with early- and intermediate-stage HCC with good liver function, TACE treatment is recommended first (56). However, TACE alone cannot lead to complete tumor necrosis (the complete necrosis rate is only approximately 20%) and has difficulty destroying peripheral tumors surrounding the tumor lesion. Repeated TACE treatments impair liver function, and therefore, locally enhanced treatments, such as local ablation, surgery, and biotherapy, are necessary to eliminate residual tumors. A repeated contrast-enhanced CT or MRI of the liver at 3 to 4 weeks after the initial TACE treatment is recommended. Subsequent combined ablation treatment can achieve a complete tumor necrosis rate of more than 90%. Repeated TACE treatments are not recommended because they can cause liver function impairment and aggravate cirrhosis. For tumors smaller than 5 cm, it is recommended to perform one TACE followed by combined ablation therapy; for tumors larger than 5 cm, subsequent combined ablation treatment can be performed after two to three TACE treatments (57, 58). On the basis of the follow-up evaluation, another TACE treatment, possibly in combination with other treatments, can be done if needed.

TARE is a trans-arterial interventional treatment in which ^90^Y or ^131^I microspheres or similar reagents are intra-arterially injected for continuous brachytherapy of cancer cells. The main indications of TARE include the following: 1. For the treatment of patients with a large tumor or multifocal or diffuse disease, which are not suitable for TACE; 2. For patients with portal vein tumor thrombus; 3. Disease progression after TACE or sorafenib treatment. Since arterial-venous or arterial-portal shunt formation are common in patients with cirrhosis, the treatment efficacy of TARE is influenced. Therefore, its application still awaits further clinical confirmation (59, 60). In China, most liver cancer is accompanied by cirrhosis, and thus, the effects of TARE on cirrhosis also need further long-term observation.

Furthermore, when TACE cannot be performed due to liver dysfunction of the patients, CTHA, CTAP, or Lp-CT can still be performed for detection of subcentimeter lesions.

### Molecular Targeted Therapy

Sorafenib and Lenvatinib, approved by the China Food and Drug Administration, are first-line treatment options for advanced HCCs (61). The STORM study suggested that adjuvant sorafenib after surgery to be ineffective (62). Retrospective studies have found that combined administration of sorafenib and TACE in the treatment of advanced liver cancer is superior to sorafenib alone (63–65); however, randomized controlled studies indicate that in European, American and Asian populations sorafenib administration on top of TACE does not improve treatment efficacy (66). The efficacy of sorafenib in adjuvant therapy after surgery, local ablation for early-stage patients, or in combination with TACE for intermediate-stage patients still awaits validation in larger cohorts of patients or prospective clinical studies.

### Radiation Therapy

Radiation therapy consists of external radiotherapy involving external irradiation of tumors and internal radiotherapy in which the radionuclide is directly implanted into the tumor or lumen invaded by the tumor. In the past, due to less developed radiotherapy equipment, radiotherapy was prone to cause radiation-induced liver disease and aggravate liver dysfunction. Moreover, most liver cancer patients with cirrhosis tolerated the treatment poorly, and thus, the application of radiotherapy in HCC was limited. Since the mid-1990s, modern, precision radiotherapy techniques (e.g., three-dimensional conformal radiotherapy, intensity-modulated conformal radiotherapy, and stereotactic radiotherapy) have developed rapidly. Radiation therapy now is mature, and have been widely used in clinical practice (67). Radioactive particle implantation dose distribution is continuously optimized, achieving satisfactory results in liver cancer treatment, especially in the treatment of portal vein tumor thrombus and hilar lymph nodes (68).

### Biological Immunotherapy

Immunotherapy for HCC primarily includes immunomodulators [interferon α, thymosin α1 (thymalfasin), etc.], immune checkpoint blockers [cytotoxic T lymphocyte-associated antigen (CTLA)-4 blocker, and programmed cell death protein 1 (PD-1) and ligand (PD-L1) blocker], tumor vaccines (dendritic cell vaccines) and cellular immunotherapy [cytokine-induced killer (CIK)]. Biotherapy can improve antitumor efficacy and enhance immunity. Phase I/II clinical trials to assess immunotherapy have found that dendritic cell treatment is safe and effective for advanced HCC (69). Studies have shown that reduction of HCC tumor volume combined with dendritic cell-CIK biotherapy can postpone the time to tumor recurrence and benefit patient survival (70, 71). Comprehensive application of CIK in cancer therapy, especially in those with clinical evaluation of complete remission and radical removal of tumor load, is considered an effective method to prevent tumor recurrence (72).

### Traditional Chinese Medicine

Many traditional Chinese medicine drugs and treatments are beneficial for reconstructing the immunity of patients, improving quality of life, reducing adverse reactions to radiotherapy and chemotherapy, and putting off tumor progression. In addition to decoction medicine, over the years, many proprietary Chinese medicines have been approved for HCC treatment, and each has their own characteristics and certain effects and has demonstrated good compliance, safety, and tolerance in patients (73). Traditional Chinese medicine advocates treatment of both the symptoms and cause of the disease. Removal of visible tumors treats the cancer-related symptoms but not the underline disease. Improving the constitutional environment and enhancing and improving immune function treat the causes and are important factors to prevent recurrence.

### Antiviral Treatment

According to the “Expert consensus of antiviral treatment of HBV/HCV-related hepatocellular carcinoma” (74), antiviral treatment for HBV/HCV-related HCC patients can reduce the recurrence and mortality of HCC, decrease HBV/HCV reactivation, control disease progression, improve liver function, and reduce the occurrence of end-stage liver disease.

### Other Treatments

Symptomatic supportive treatments primarily include analgesics, liver protection, cholagogues, improvement of nutritional status, correction of anemia and hypoproteinemia, control of ascites or pleural effusion, and prevention and treatment of gastrointestinal hemorrhage. These symptomatic supportive treatments can alleviate patient suffering, improve quality of life, ensure smooth progression of anticancer treatments, and even improve treatment efficacy or provide opportunity for further treatment.

### Comprehensive Treatment Principles and Multidisciplinary Comprehensive Treatment

**Figure 2:**
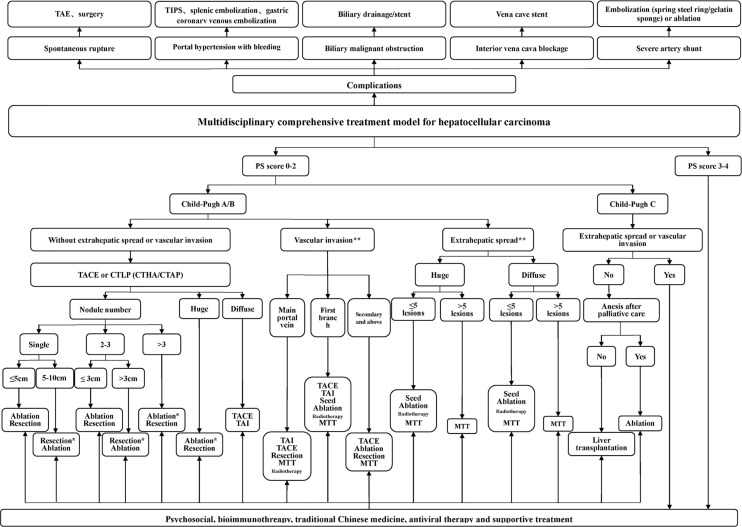
Multidisciplinary, comprehensive treatment model. TIPS: transjugular intrahepatic portosystemic shunt; PS (performance status) score: systemic condition test score; Massive type: maximum tumor diameter > 10 cm; Diffuse type: extensive and diffusely distributed intrahepatic cancer lesions with unclear borders, often accompanied by cirrhosis; Ablation: RFA, MWA, and PEI, among others, but primarily RFA and MWA; *Efficacy comparison of surgery and TACE combined with ablation awaits confirmation in further randomized multicenter clinical studies. **Treatment of portal vein invasion and extrahepatic metastasis should be based on control of intrahepatic lesions, which mostly requires multidisciplinary, comprehensive treatment. 1. Tumors in the safe area are preferably ablated. When a tumor is in a dangerous area and is not suitable for ablation, surgery is considered. 2. Ablation treatment can be repeated. For a tumor that is difficult to ablate in one treatment, the residual portion can be ablated again after follow-up examinations.

The proposed treatment algorithm of HCC is shown in **Figure 2**.

## Eight Highlights of the Consensus

### Hepatic Angiography, CTHA, CTAP, Lp-CT, and TACE-CT Are Useful in Detection of Focal Liver Lesions and Accurate Staging

**Figure 3:**
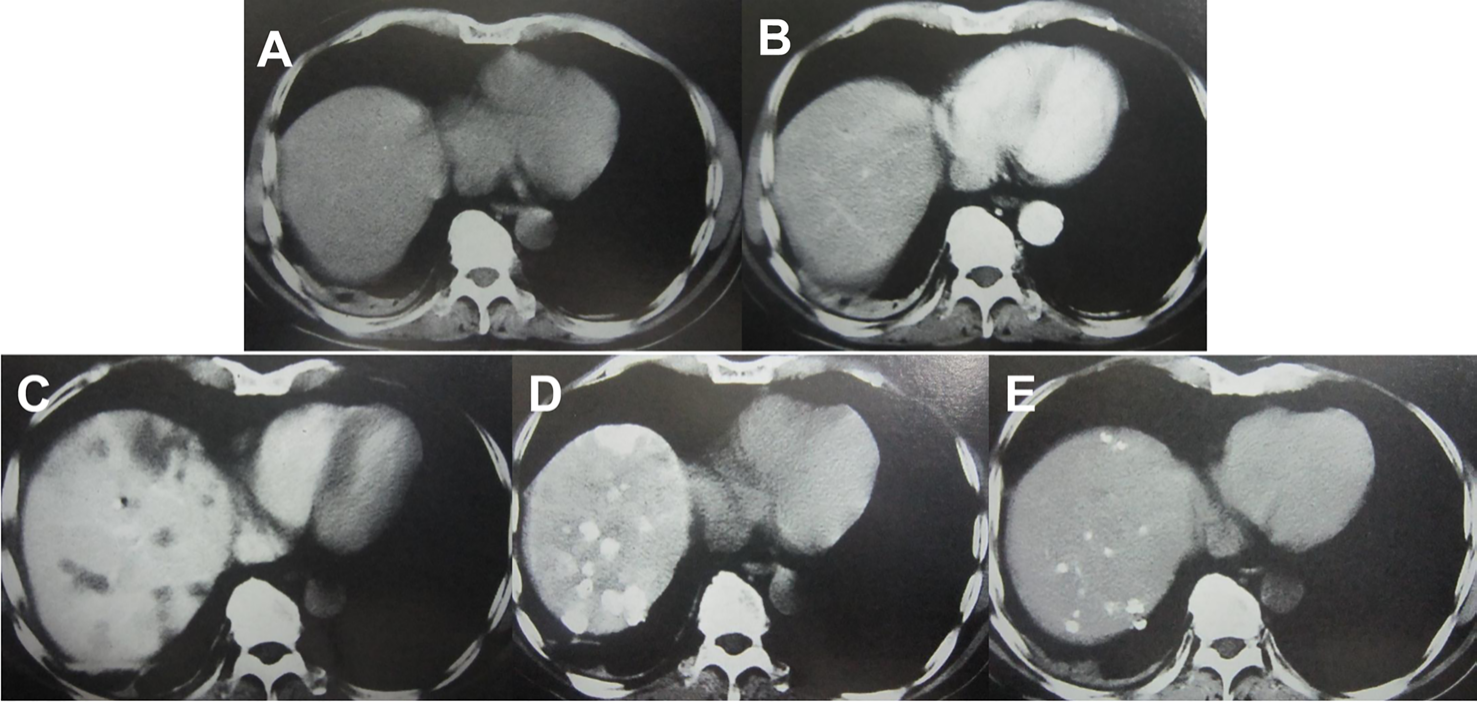
Imaging examination of an HCC patient with a progressively elevated AFP level. **(A)** CT plain scan showed no lesions in the liver; **(B)** A contrast-enhanced CT scan failed to reveal any lesions in the liver; **(C)** CTAP showed intrahepatic low-density lesions; **(D)** CTHA showed intrahepatic high-density lesions; **(E)** Lp-CT showed multiple intrahepatic lipiodol deposits after 3 weeks.

**Figure 4:**
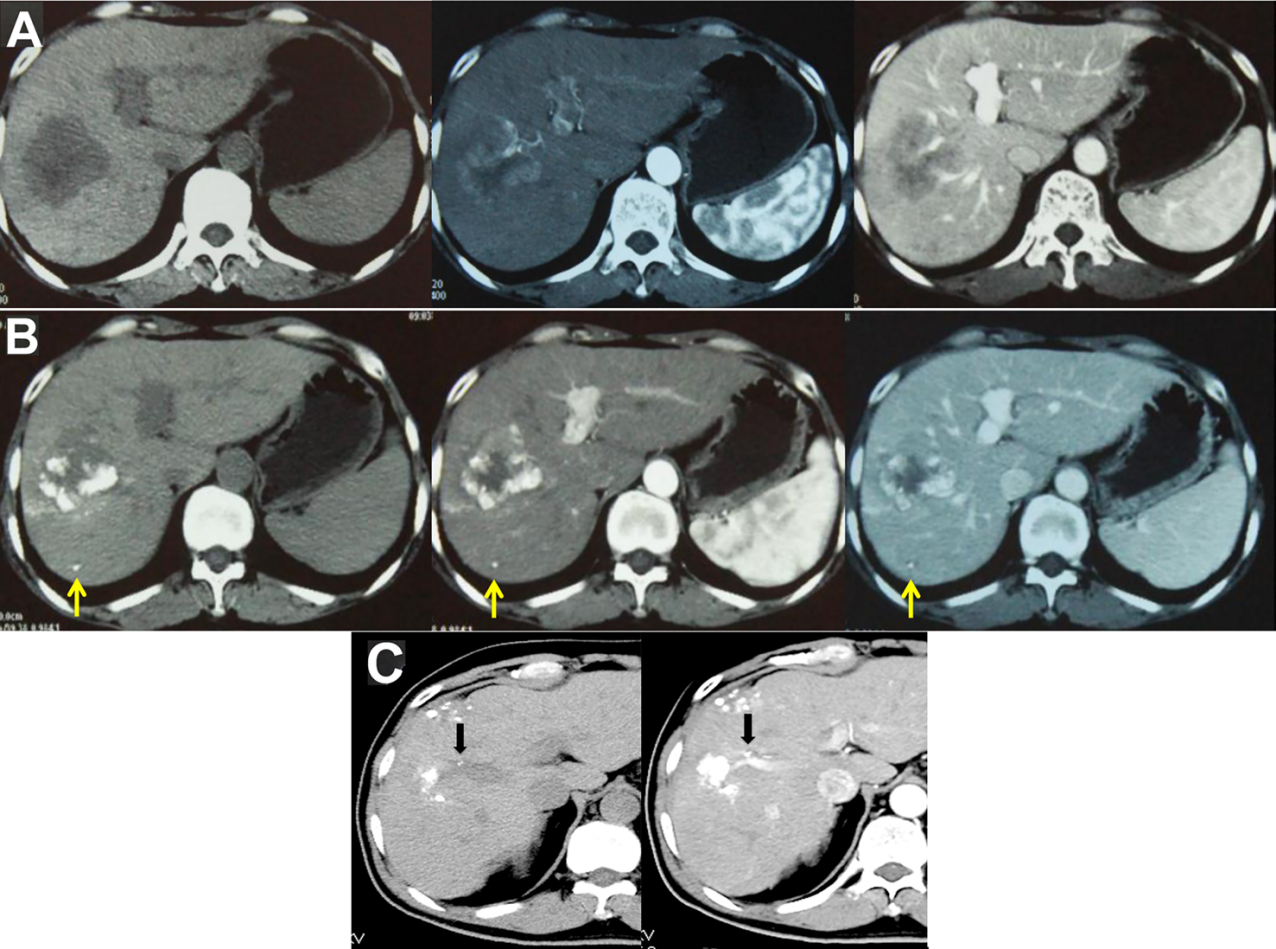
TACE-CT enables detection of focal loci and precise staging. **(A)** CT before treatment showed a 7-cm tumor in the S5 and S6 region of the liver; **(B)** Follow-up CT examination at 4 weeks after TACE showed that the tumor volume at S5 and S6 was reduced, while a small lesion was detected at S6, as indicated by the arrow; **(C)** In another male patient, follow-up CT examination at 3 weeks after TACE showed intravascular lipiodol deposits (indicated by the arrow), suggesting the formation of an intravascular small tumor thrombus.

Imaging examinations play an important role in HCC diagnosis, and typical imaging characteristics indicating HCC are arterial phase enhancement and washout during the portal venous phase and delayed phase on CT or MRI, which have been included in the guidelines in different countries worldwide (75–78). However, the sensitivity of imaging examinations is limited, especially in the diagnosis of small HCC lesions. Moreover, HCC has multicenter origins and often exhibits early invasion of the small branches of the portal vein, intrahepatic metastasis, and high incidence of recurrence after liver resection. Therefore, early detection of small lesions in the liver and accurate evaluation of focal liver lesions and intrahepatic metastasis are important for disease treatment and prognosis assessment. Hepatic angiography, CTHA, CTAP, Lp-CT, and TACE-CT are useful in detection of focal liver lesions, accurate staging and planning of a suitable treatment regimen (**Figures 3** and **4**). TACE-CT can detect approximately 15% of lesions (most of which are < 5 mm or even < 3 mm) that are unrecognizable in a conventional CT scan (22–26).

### TACE/Ablation as the First Choice for Treatment of Early-Stage HCC

The 2019 National Comprehensive Cancer Network (NCCN) guidelines recommend surgical resection or topical treatment for early-stage liver cancer (75). The third edition of the 2019 Japanese Liver Disease Association recommends surgical resection for patients with a single tumor and grade A or B liver function and further recommends RFA as an alternative regimen for patients with tumors smaller than 3 cm (77). The 2018 edition of the European Association of Liver Research guidelines recommend RFA for very early-stage liver cancer (BCLC stage 0) and surgical resection, liver transplantation and ablation treatment for early-stage liver cancer (BCLC stage A) (76). The 2019 edition of China specifications for diagnosis and treatment of primary liver cancer lists surgical resection and ablation as treatment options for early-stage liver cancer (78). Therefore, currently, whether surgical resection or ablation is the preferred treatment for early-stage liver cancer is still controversial.

At present, some clinical studies compared RFA and surgical resection in the treatment of a single small HCC (31, 32, 37, 79–83), and the controversial results remained. Peng et al. (37) enrolled 145 patients with early-stage HCC, of whom 71 underwent RFA and 74 underwent surgical resection. They found that RFA was superior in efficacy and safety to surgical resection, especially when the tumor lesions were more than 3 cm away from the Glisson’s capsule. Liu et al. (79) found that among 79 patients with RFA and 79 patients with surgical resection, tumor recurrence and survival were better in the surgical group than in the RFA group and concluded that surgical resection should be considered the preferred treatment method. Chen et al. (80) conducted a prospective, randomized, controlled study of patients with HCC ≤ 5 cm; 71 patients received RFA, and 90 patients underwent surgical resection. There was no survival difference between the two groups of patients, and subgroup analysis based on tumor size (≤ 3 cm and 3 to 5 cm) showed similar results. Therefore, the authors concluded that RFA, which is less invasive, had an efficacy similar to that of surgical resection. Huang et al. (81) compared RFA and surgical treatment for HCC patients with a single lesion <3 cm and found no difference in tumor control and survival between the 121 patients who underwent ablation and the 225 patients who underwent surgical resection, and the quality of life score in the ablation group was significantly better than that in the surgery group. Kang et al. (82) compared 198 patients with early-stage HCC using propensity-matching analysis and reached a similar conclusion that the efficacy of RFA treatment was comparable to that of surgical resection, and patients with RFA had fewer complications, faster recovery, and a significantly shortened hospitalization time. Kutlu et al. (83) used the US National Cancer Institute’s “surveillance, epidemiology, and end results (SEER) database” to conduct a large-scale study of 1,894 patients with HCC between 2004 and 2013 and found that there was no significant difference in the efficacy of RFA and surgical resection for tumors < 3 cm. Majumdar et al. (32) systematically reviewed four clinical studies that included 574 patients to compare RFA with surgical resection and found that there was no significant difference in survival between the two treatments. In summary, ablation and surgery have similar efficacy in the treatment of early-stage HCC, and ablation is more advantageous from a health economics perspective (84). Therefore, the authors emphasize prioritizing interventional ablation treatment and only suggest surgical resection under circumstances when ablation is not appropriate.

**Figure 5:**
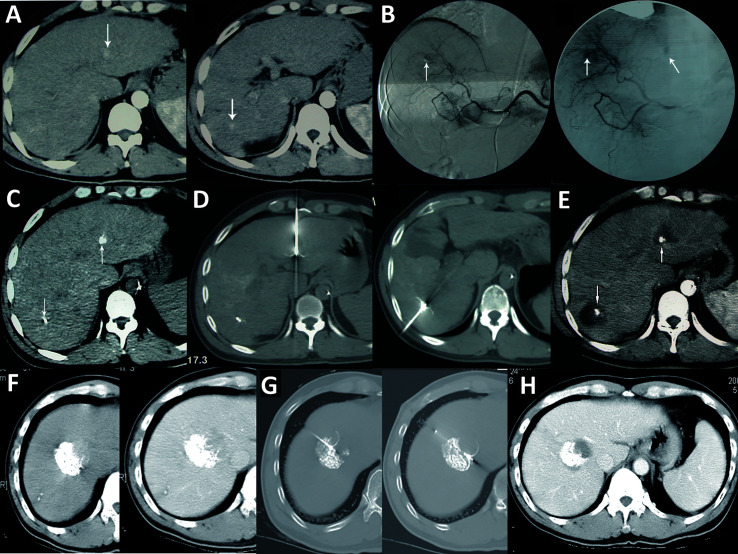
TACE combined with ablation is the preferred treatment for HCC at an ideal location. **(A)** A case of HCC with a progressively increased AFP level. Liver CT shows two lesions in the left lateral segment and right posteroinferior segment of the liver; **(B)** DSA during the TACE treatment showed staining of tumors in the left and right lobe (arrow); **(C)** Lp-CT confirmed two lesions; **(D)** CT-guided radiofrequency ablation of lipiodol deposited lesions; **(E)** One month after combined treatment, AFP decreased to a normal level, and no tumor recurrence was observed five year later in the follow-up CT scan. **(F)** Another case of HCC after TACE treatment combined with radiofrequency ablation treatment **(G)**; **(H)** A 15-year postoperative follow-up examination showed complete inactivation of the lesion.

TACE is a holistic treatment at the organ level and is applied at the first step of minimally invasive treatment. Its main role is to reduce tumor blood supply and reduce tumor load. Emulsified lipiodol and chemotherapeutic drugs are deposited in small lesions or foci that are difficult to detect by conventional contrast-enhanced CT scanning, thereby treating small lesions or foci while also revealing them and guiding the next step in minimally invasive treatment. TACE combined with ablation has demonstrated a higher survival rate and better tumor control rate than simple ablation and does not significantly increase the incidence of complications (85–87). Therefore, TACE/ablation has obvious advantages as a primary treatment for early-stage HCC (**Figure 5**).

### Infiltrative HCC as an Independent Subtype

**Figure 6:**
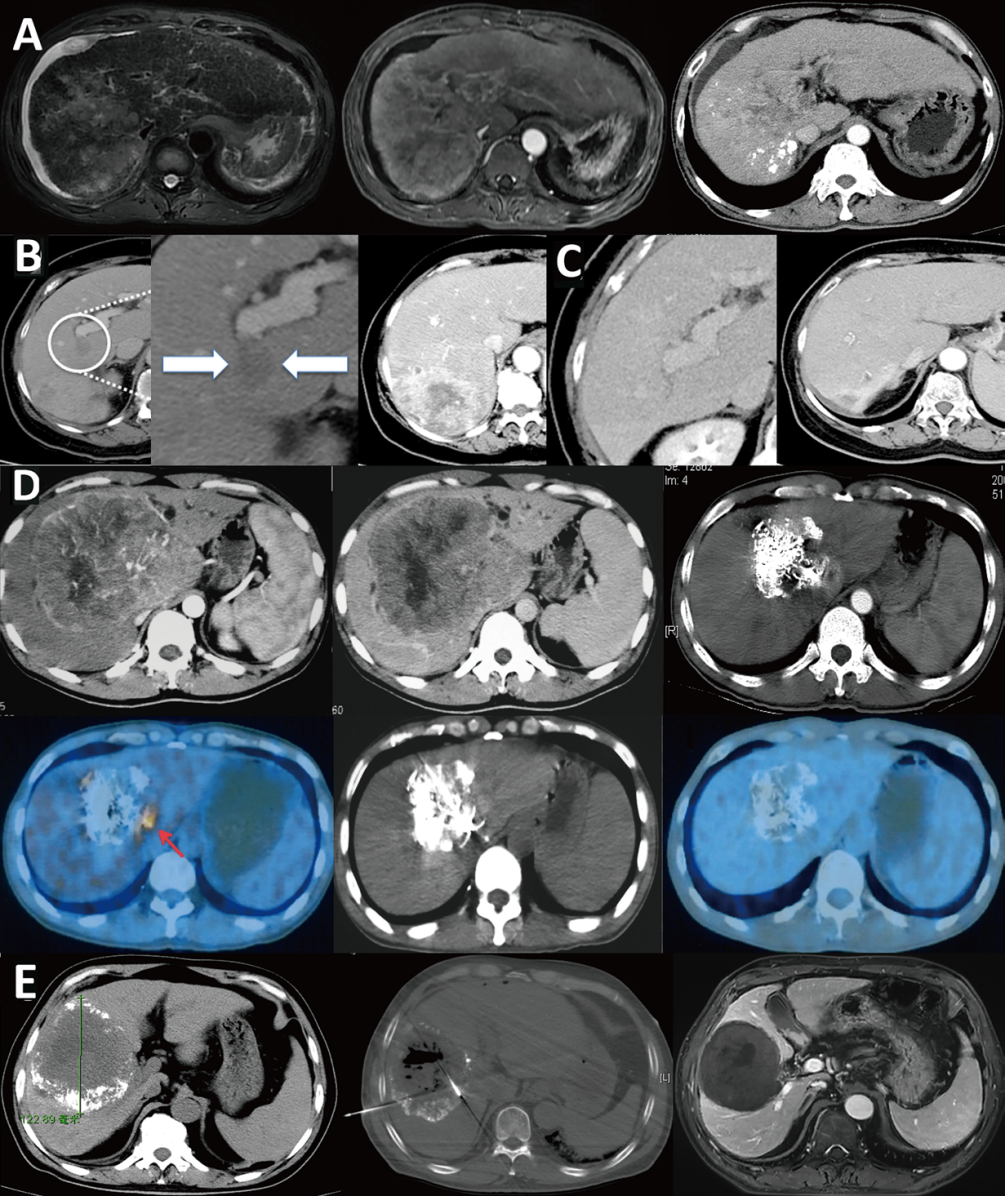
Infiltrative HCC is different from massive HCC and should be classified separately. **(A)** Infiltrative HCC in a patient with hepatitis B cirrhosis. No obvious boundary around the tumor, combined with tumor thrombus in the portal vein and its branches. After TACE treatment, scattered lipiodol was deposited. The treatment effect and prognosis were poor; **(B)** Massive HCC in a patient with a large16-cm liver tumor in the right lobe that has an intact tumor capsule and clear boundary. After three TACE treatments, the tumor was significantly reduced to 8 cm. Follow-up PET/CT examination showed a residual active portion at the edge of the tumor, and another ablation treatment was performed. Follow-up checks showed no tumor activity, and complete remission was achieved. The patient has survived for 16 years and is still alive; **(C)** Another massive liver cancer lesion in the right lobe of a patient. After three TACE treatments combined with microwave ablation, the tumor was completely inactivated; **(D)** Infiltrative HCC patient with an unclear tumor boundary and right portal vein tumor thrombus; **(E)** The tumor and tumor thrombus were significantly reduced after nine courses of HAIC.

Infiltrative HCC is divided into diffuse and nondiffuse types. The imaging manifestation of infiltrative HCC is a lack of a clear boundary between tumor and normal liver tissue. Patients often have a history of cirrhosis. This HCC type accounts for approximately 7% to 13% of total HCC cases (14, 15). Risk factor analysis has shown that infiltrative HCC is more common in patients with HBV infection (88). Moreover, this type of HCC has diffuse distribution characteristics and is more likely to invade the portal vein system. Statistically, portal vein invasion in infiltrative HCC is much more common than in the noninfiltrating type (68% *vs*. 25%, P < 0.001) (14), and the prognosis of infiltrative HCC is worse than that of nodular type HCC with an intact capsule (**Figure 6**). Benvegnu et al. (88) reported that the 1-year and 3-year survival rates for HCC patients with an intact capsule were 75.4% and 46.0%, respectively, while those for infiltrative HCC patients were 33.3% and 13.6%, respectively. Kneuertz et al. (14) reported that the 1-year and 3-year survival rates for infiltrative HCC patients were 43% and 29%, respectively, and the median survival time was only 10 months. The treatment options for infiltrative HCC are also limited because the majority of patients are in the advanced stage, and tumor infiltration and growth are typically accompanied by portal vein invasion, which is a contraindication for surgical resection and liver transplantation. Transarterial intravascular treatment is an effective and feasible treatment for infiltrative HCC. Studies conducted by Lyu et al. (89, 90) indicated that HAIC for patients with advanced HCC accompanied by portal vein tumor thrombus was effective. A phase II, single-arm clinical trial showed that 49 patients achieved a good tumor control rate, a considerable survival rate, fewer adverse reactions, and a higher quality of life after treatment. Further comparison with administration of oral sorafenib, a targeted drug, showed that the median progression-free survival time of the HAIC group was longer than that of the sorafenib group [based on the modified response evaluation criteria in solid tumors (mRECIST), 7.4 months *vs*. 3.6 months, P< 0.001] and the median survival time was better than that of the sorafenib group (14.5 months *vs*. 7.0 months, P < 0.001). In the meantime, a study of 147 propensity matched pairs further confirmed the reliability of the results. Multivariate regression analysis also confirmed that HAIC was a favorable factor for tumor control (P < 0.001) and extended survival time (P < 0.001) of patients. For specific protocols for hepatic arterial infusion chemotherapy, refer to the literature (91).

### Minimally Invasive and Comprehensive Treatment of Metastatic Lymph Nodes

**Figure 7:**
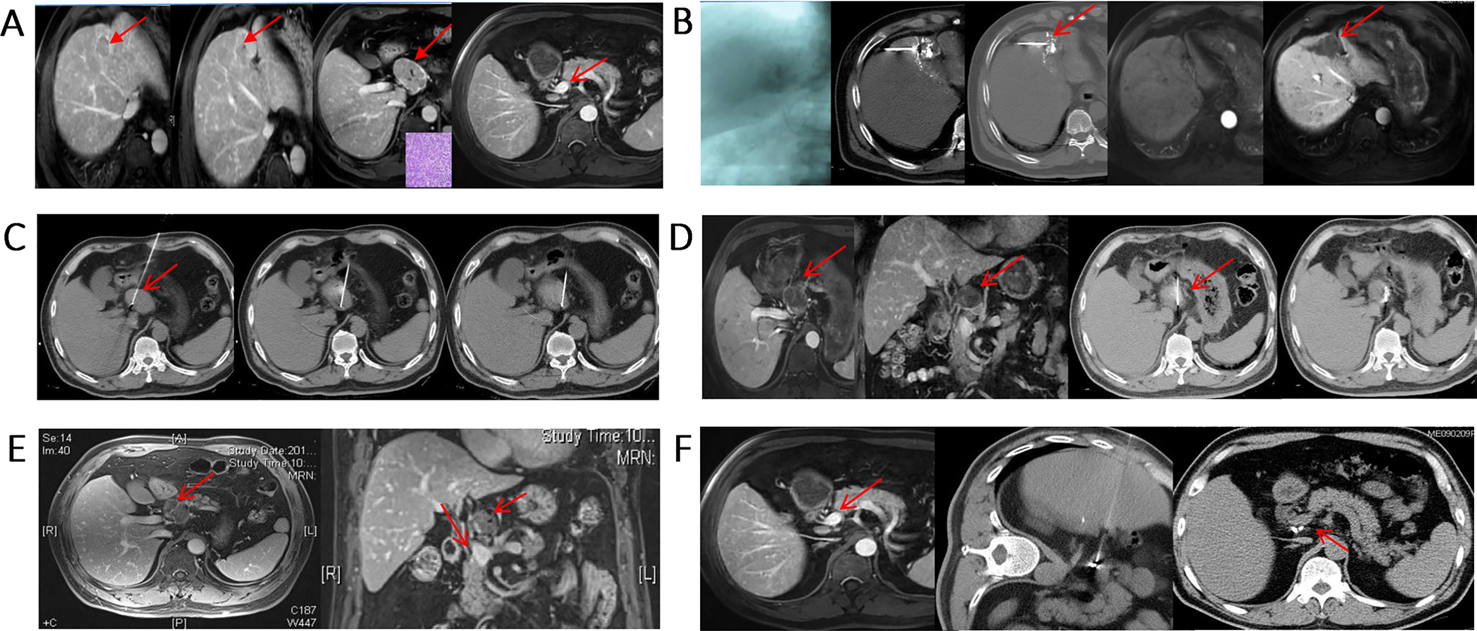
Minimally invasive, comprehensive treatment for metastatic lymph nodes. **(A)** S4 HCC with metastatic lymph nodes in the portal vena cava space that were pathologically confirmed as HCC. The AFP level was 439 ng/mL before treatment; **(B)** Two weeks after TACE treatment, subsequent RFA was performed; **(C)** RFA ablation of hepatic portal metastatic lymph nodes; **(D)** Lymph nodes were basically inactivated, and 125I particles were implanted in the remaining portion; **(E)** Metastatic lymph nodes were completely necrotic, and the AFP level decreased to 14 ng/mL; **(F)** After adjuvant CIK treatment, the metastatic lymph nodes were completely inactivated, and AFP was further reduced to 2 ng/mL. The patient is still alive 10 years after treatment.

In advanced patients with extrahepatic metastases *via* molecular targeted therapy (sorafenib), although effective, extends survival time by only 3 months. Furthermore, patients at this stage usually die of intrahepatic lesion progression rather than extrahepatic metastasis. Therefore, multidisciplinary comprehensive therapy consisting of treatment of intrahepatic lesions combined with local treatment of extrahepatic metastases is still advocated (92–97) (**Figure 7**). The principles of treatment are as follows: 1. Protection of lymph nodes with normal function; 2. Inactivation of metastatic lymph nodes; 3. Close observation and follow-up of suspect lymph nodes.

### Multilevel Subdivision of M Staging Used for Guiding Treatment and Predicting Prognosis

**Figure 8:**
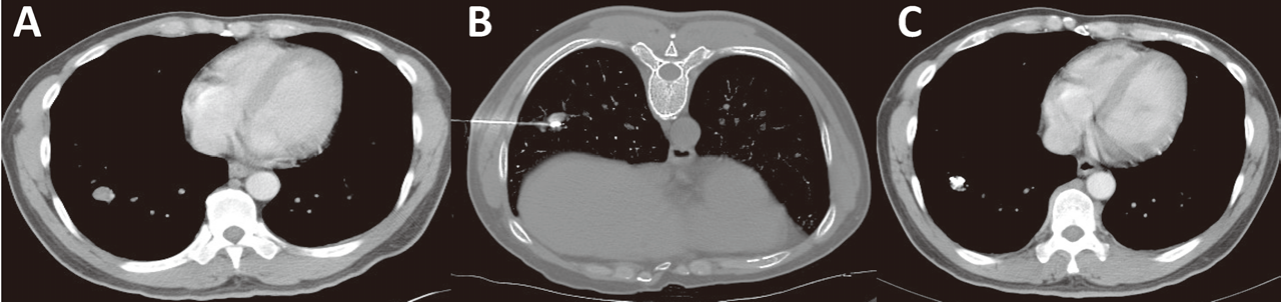
Minimally invasive treatment for HCC with pulmonary oligo-metastasis. **(A)** CT of an HCC patient showing newly developed right lung oligo-metastatic lesions. **(B)** Percutaneous particle implantation was performed. **(C)** A 6-month follow-up CT showed that the metastatic tumor had shrunk and exhibited no activity.

Stage IV HCC should be subdivided to distinguish patients with limited metastasis from patients with a heavy metastatic tumor load. For example, patients with a single metastatic lesion in a single metastatic organ are defined as M1-1, patients with multiple metastatic lesions in a single metastatic organ are defined as M1-m, and so on (**Table 1**). For patients at different stages, limited metastatic lesions can be eliminated by ablation or particle implantation, resulting in longer survival (93, 94, 97) (**Figure 8**), rather than treated by administration of a molecular targeted drug alone as suggested in the guidelines.

**Table 1:** Multilevel subdivision of M staging.

M staging	Criteria
M1-1	1 organ, 1 metastatic lesion
M1-2	1 organ, 2 metastatic lesions
M1-3	1 organ, 3 metastatic lesions
M1-4	1 organ, 4 metastatic lesions
M1-m	1 organ, multiple metastatic lesions
M2-2	2 organs, 2 metastatic lesions
M3-3	3 organs, 3 metastatic lesions
Mm-m	> 3 organs, ≥ 5 metastatic lesions

M, metastasis; m, multiple.

### HCC Patients With Severe Hepatic Decompensation Are Candidates for Liver Transplantation

In recent years, immunotherapy has become a research hotspot and is the future trend in cancer treatment. The 2018 Nobel Prize in Medicine was awarded to two cancer immunotherapy scientists. Tumor immunotherapy is a treatment method for controlling and clearing tumors by reactivating and maintaining the recognition and killing abilities of the immune system toward tumor cells and restoring the normal antitumor immune responses of the body. Some studies suggested that future cancer treatment should be combined with immunotherapy rather than employ a single treatment regimen (98). The CheckMate 040 phase I/II phase clinical trial validated the efficacy and safety of the anti-PD-1 antibody nivolumab in patients with advanced HCC (99). In March 2020, the US Food and Drug Administration (FDA) approved nivolumab plus ipilimumab for HCC previously treated with sorafenib (100). However, liver transplant patients, who suffer from tremendous surgical trauma, need relatively longer recovery time, require many economic and medical resources, and still have issues of recurrence and metastasis. In the meantime, long-term use of immunosuppressive agents is required after surgery, and thus, the valuable opportunity for immunotherapy is missed for many patients, which conflicts with the future direction of cancer treatment.

### Promotion of Bioimmunotherapy, Traditional Chinese Medicine Therapy, Antiviral Therapy, and Psychosocial and Psychopharmacological Interventions, Which Should Be Involved in All Stages of Treatment

**Figure 9:**
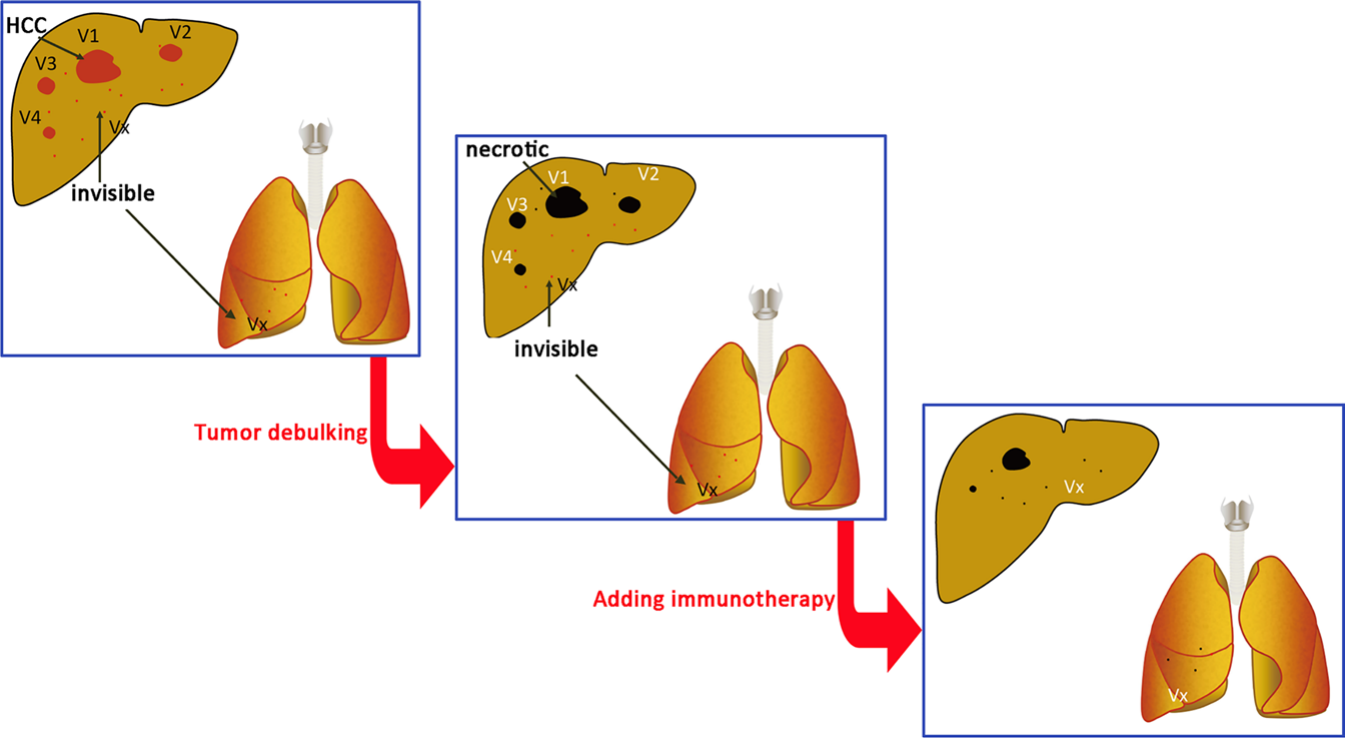
Immune cell treatment performed at the same time or after tumor debulking. V1, V2, V3…Vn are radiographically visible intrahepatic tumors. Vx is a lesion that has not been detected by intrahepatic or extrahepatic imaging examination. The total tumor burden (volume total, VT) = V1 + V2 + V3… + Vn + Vx. The goal of treatment is to reduce the tumor burden to VT = Vx and then use immune cells to treat Vx.

Liver cancer requires treatment of both the symptoms and the underlying basic causes. Direct treatment of tumors is treatment of the symptoms, and protection of a patient’s biological immune functions and establishment of excellent psychosocial support treat the underlying basis. HCC is an inflammation-related cancer, and studies have confirmed that immune remission is associated with tumor and patient outcome (101). Biotherapy can strengthen a patient’s immunity and ultimately improve antitumor effects (**Figure 9**). Current immuno-therapeutic strategies are based on two fundamental principles: 1. The ability to evoke current immune responses; 2. The need to stimulate new or different immune responses. Unleashing current immune response relies on reactivity of a pre-existing immunity to cancer which is restricted by micro-environmental factors, such as inhibitory receptors on T cells especially PD-1 and CTLA-4, or alternatively immunosuppressive cytokines such as TGF-β. Checkpoint inhibitors fall within this category. Conversely, antibodies that directly target molecules expressed on HCC, such as alpha- AFP, are within the second category. These strategies can be enhanced by coupling these antibodies to effector cells, such as T cells or even NK cells. The first-line checkpoint inhibitors approved for use in HCC by NCCN (75) are as following: Atezolizumab + bevacizumab is preferred regimens, while Nivolumab is applicable if patient is ineligible for tyrosine kinase inhibitors [TKIs] or other anti-angiogenic agents. As subsequent-line therapy if disease progression, Nivolumab, Nivolumab + ipilimumab, Ramucirumab, and Pembrolizumab are optional.

Efficient combination of the various drugs and treatment methods provided by traditional Chinese medicine can enhance the body’s immunity, reduce adverse reactions to radiotherapy and chemotherapy, and improve the quality of life of patients. In addition to decoction medicine, the China drug regulatory authorities have approved several modern Chinese medicine preparations for HCC treatment (102), and all have demonstrated unique characteristics and certain effects, with good patient compliance, safety and tolerance, and thus can be used as appropriate. Sustained infection with HBV and/or HCV is an important risk factor for HCC development, progression, and recurrence. Antiviral therapy is very important in liver cancer patients with HBV infection and active replication of the virus. Antiviral therapy can reduce the rate of postoperative recurrence (103, 104). Therefore, antiviral therapy should be involved throughout the liver cancer treatment process. The mental status of patients with liver cancer and their families should be considered, and effective measures should be employed to help them face the disease positively and to reduce depression, fear, and anxiety.

However, the treatment strategy for clinical patients should be made according to their own characteristics, so that patients can get the most benefit from treatment. Bioimmunotherapy, traditional Chinese medicine therapy, antiviral therapy, and psychosocial and psychopharmacological interventions cover a very wide area involving multidisciplinary approach. Therefore, determination of appropriate therapy should include a careful patient/physician discussion. Many clinical trials on combined treatments for HCC are ongoing, including assessing combinations with immune checkpoint inhibitors. Further clinical evidences are required to explore the reliable treatment schedule, which might allow a more precise selection of treatment in well-defined patients.

### Implementation of Multicenter, Randomized, Controlled Studies of Minimally Invasive Treatment and Surgery for Early- and Intermediate-Stage HCC

In choosing treatment methods for early- and intermediate-stage HCC, there are still major differences in different countries and in different disciplines (96), and multicenter, randomized, controlled studies are lacking. For example, in the newly published China Liver Cancer 2019 guidelines, there are three treatment options for early-stage liver cancer: surgery, ablation, and liver transplantation. These three completely different treatment methods confuse both patients and doctors. The aforementioned questions still await verification in additional large-scale multicenter randomized controlled studies. Therefore, conducting multicenter, randomized, controlled clinical studies of minimally invasive and surgical treatments for early- and intermediate-stage HCC is recommended.

## Data Availability Statement

The original contributions presented in the study are included in the article/supplementary material. Further inquiries can be directed to the corresponding author.

## Author Contributions

All authors discussed the recommendations. Q-FC, WL, and PW developed the first outline. All the authors were involved in revising when the first draft had been developed. All the authors refined the contents with feedbacks, and comments. All authors contributed to the article and approved the submitted version.

## Conflict of Interest

The authors declare that the research was conducted in the absence of any commercial or financial relationships that could be construed as a potential conflict of interest.
